# Gymnosperms of Idaho: Chemical Compositions and Enantiomeric Distributions of Essential Oils of *Abies lasiocarpa*, *Picea engelmannii*, *Pinus contorta*, *Pseudotsuga menziesii*, and *Thuja plicata*

**DOI:** 10.3390/molecules28062477

**Published:** 2023-03-08

**Authors:** Kathy Swor, Prabodh Satyal, Ambika Poudel, William N. Setzer

**Affiliations:** 1 Independent Researcher, 1432 W. Heartland Dr., Kuna, ID 83634, USA; 2 Aromatic Plant Research Center, 230 N 1200 E, Suite 100, Lehi, UT 84043, USA; psatyal@aromaticplant.org (P.S.);; 3 Department of Chemistry, University of Alabama in Huntsville, Huntsville, AL 35899, USA

**Keywords:** subalpine fir, Engelmann spruce, lodgepole pine, Douglas fir, western red cedar, gas chromatography, chiral

## Abstract

Conifers are of great economic value in terms of lumber production, important for construction and other uses such as pulp and paper. They are also important sources of essential oils. Conifer species have been vital to the ethnobotany and traditional herbal medicine of many different Native American groups. The objective of this work was to obtain and analyze the essential oils of several conifer species (*Abies lasiocarpa*, *Picea engelmannii*, *Pinus contorta*, *Pseudotsuga menziesii*, and *Thuja plicata*) growing in Idaho. The foliar essential oils were obtained by hydrodistillation and then analyzed by gas chromatographic methods, including GC-MS, GC-FID, and chiral GC-MS. The essential oils were obtained in varying yields from 0.66% up to 4.70%. The essential oil compositions were largely dominated by monoterpene hydrocarbons and oxygenated monoterpenoids. The chiral monoterpenoids were generally rich in the (−)-enantiomers for members of the Pinaceae, but the (+)-enantiomers predominated in the Cupressaceae. The essential oil compositions obtained in this work are qualitatively similar, but quantitatively different, to previously reported compositions and confirm and complement the previous reports. However, this is the first comprehensive analysis of the chiral terpenoid components in these conifer species. Additional research on essential oils of the Pinaceae and Cupressaceae is needed to describe the chemical profiles, chemical compositions, and enantiomeric distributions more reliably in the various species and infraspecific taxa of these two families.

## 1. Introduction

Idaho, and western North America in general, is home to great habitat diversity, including mountains, canyons, and Great Basin deserts, and is also home to a large number of conifer species. Many of these trees are important sources of timber and other forest products; they have been important in Native American cultures in traditional medicine, and in addition to wood and wood products, are sources of essential oils. As part of our ongoing investigation into the essential oils of Idaho, we have collected samples of Rocky Mountain subalpine fir (*Abies lasiocarpa* var. *lasiocarpa*) (Pinaceae), Engelmann spruce (*Picea engelmannii* subsp. *engelmannii*) (Pinaceae), Rocky Mountain lodgepole pine (*Pinus contorta* subsp. *latifolia*) (Pinaceae), Rocky Mountain Douglas fir (*Pseudotsuga menziesii* var. *glauca*) (Pinaceae), and western red cedar (*Thuja plicata*) (Cupressaceae) growing in Idaho. The foliar essential oils have been obtained by hydrodistillation and the essential oils analyzed by gas chromatographic (GC-MS and GC-FID) methods. The enantiomeric distributions of monoterpenoid components have also been examined using chiral GC-MS.

**Figure 1:**
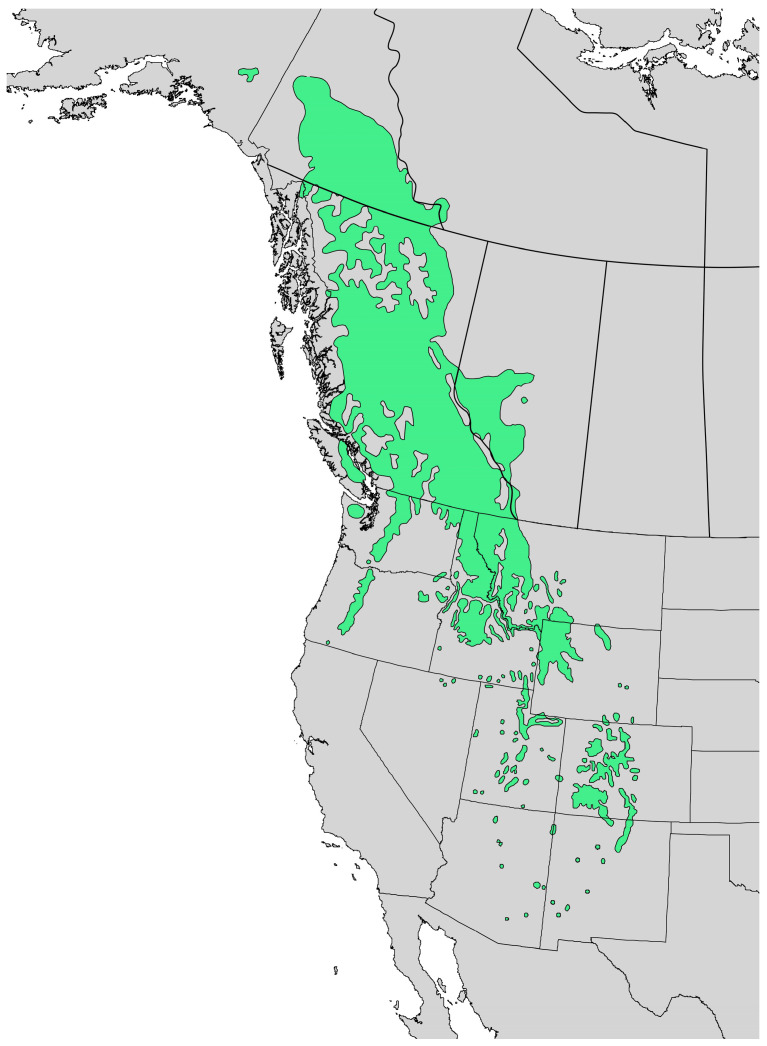
Natural range of *Abies lasiocarpa* [6].

**Figure 2:**
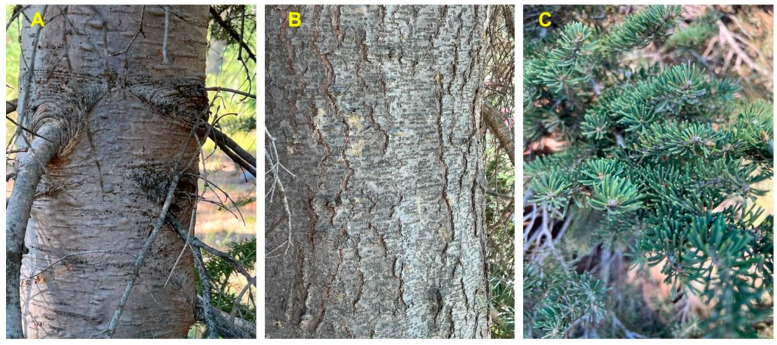
*Abies lasiocarpa* var. *lasiocarpa* from southern Idaho. (**A**): bark of young tree. (**B**): bark of old tree. (**C**): foliage. Photographs by K. Swor.

*Abies lasiocarpa* (Hook.) Nutt. (subalpine fir, Pinaceae) is native to the mountains of western North America (Figure 1) [1]. On young trees, the bark is smooth and gray with resin blisters, but appears rough and fissured on older trees. The leaves are flat needles, 1.5–3 cm long (Figure 2). The infraspecific taxonomy of *A. lasiocarpa* has been debated and three varieties have been suggested: *Abies lasiocarpa* (Hook.) Nutt. var. *lasiocarpa* (coastal subalpine fir, ranging from British Columbia south through the Cascade Mountains of Washington and Oregon); *Abies lasiocarpa* var. *bifolia* (A. Murray bis) Eckenw. (Rocky Mountain subalpine fir, ranging from British Columbia south through the Rocky Mountains of Idaho, Montana and Colorado); and *Abies lasiocarpa* var. *arizonica* (Merriam) Lemmon (corkbark fir, found in high mountains of Arizona and New Mexico) based on morphological and monoterpenoid profiles [2,3]. However, based on DNA data, there is little support for the recognition of *A. l.* var. *bifolia* as a distinct variety, but rather a chemotype of *A. l.* var. *lasiocarpa* due to geographical selection differences [2]. The foliar essential oil compositions of the three varieties have been investigated previously by Hunt and von Rudloff [4] and by Adams and co-authors [2]. The essential oil of coastal subalpine fir has been characterized by relatively high concentrations of β-phellandrene (36.8–58.8%), while Rocky Mountain subalpine fir essential oil is rich in camphene (7.3–16.2%) and bornyl acetate (13.0–31.6%) [4]. Corkbark fir also has high concentrations of camphene (15.2%) and bornyl acetate (34.4%) [2]. The Shoshoni people took an infusion of the needles of *A. lasiocarpa* to treat colds [5].

**Figure 3:**
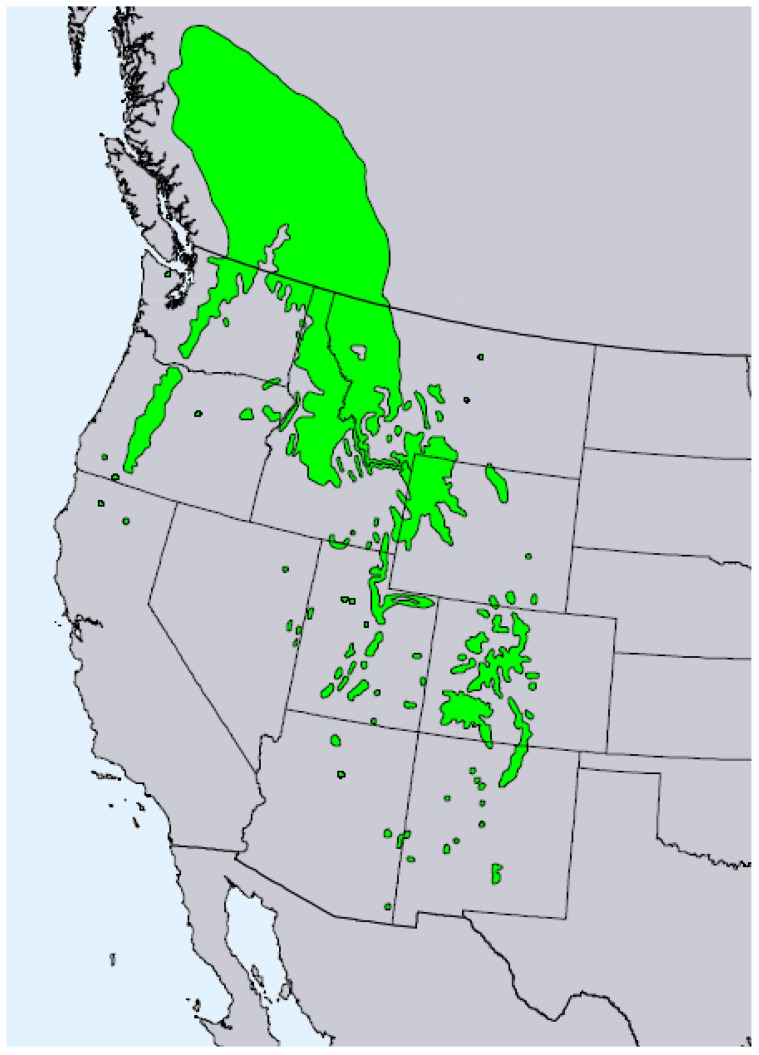
Natural range of *Picea engelmannii* [6].

**Figure 4:**
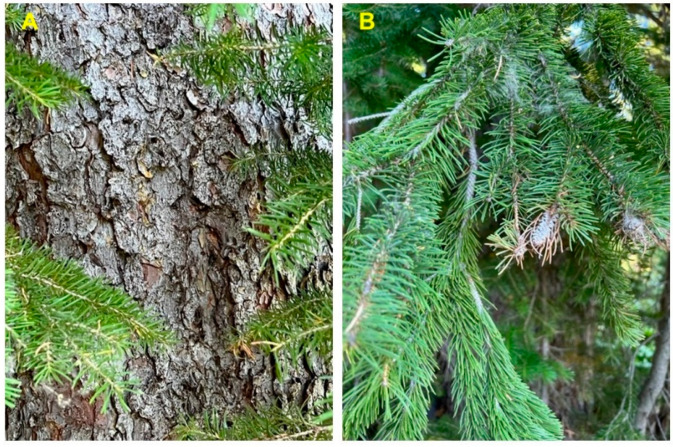
*Picea engelmannii* subsp. *engelmannii* from southern Idaho. (**A**): bark. (**B**): foliage. Photographs by K. Swor.

*Picea engelmannii* Engelm. (Engelmann spruce, Pinaceae) is widely distributed in western North America and ranges from British Columbia and Alberta, Canada, south through the Cascade Mountains of Washington and Oregon, and through the Rocky Mountains of Idaho, Montana, Wyoming, Colorado, and New Mexico, as well as Utah and Arizona (Figure 3) [7]. Two subspecies of *P. engelmannii* have been recognized [8], *P. engelmannii* subsp. *engelmanii* and *Picea engelmannii* subsp. *mexicana* (Martínex) P.A. Schmidt, which is found on the high mountains of northern Mexico [9]. The bark of *P. engelmannii* is thin and flaky; the needles are 15–30 mm long (Figure 4).

**Figure 5:**
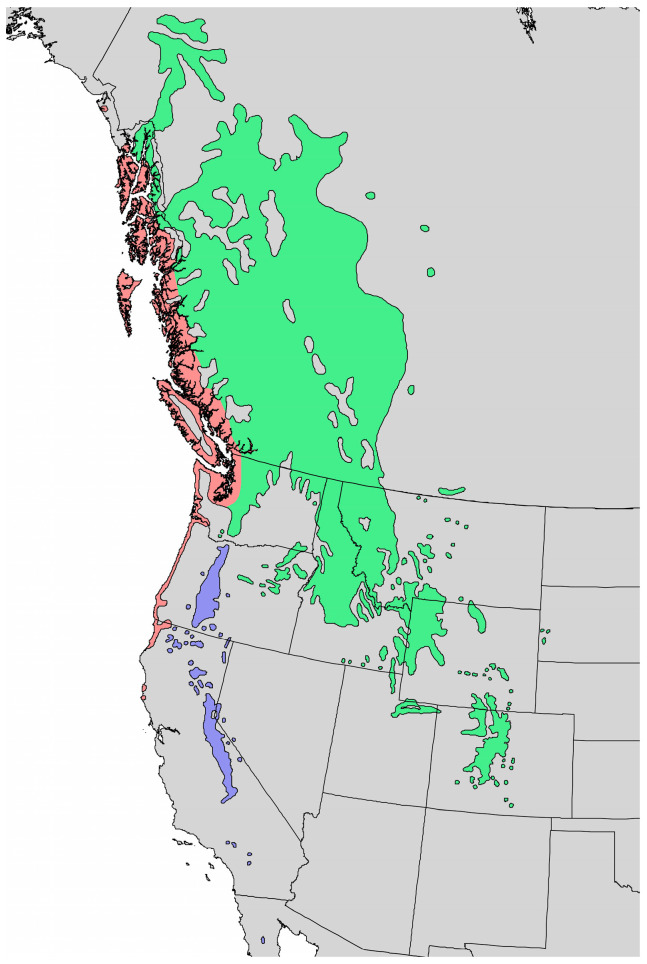
Natural range of *Pinus contorta*. 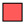 *P. contorta* subsp. *contorta*. 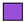 *P. contorta* subsp. *murrayana*. 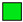 *P. contorta* subsp. *latifolia* [6].

**Figure 6:**
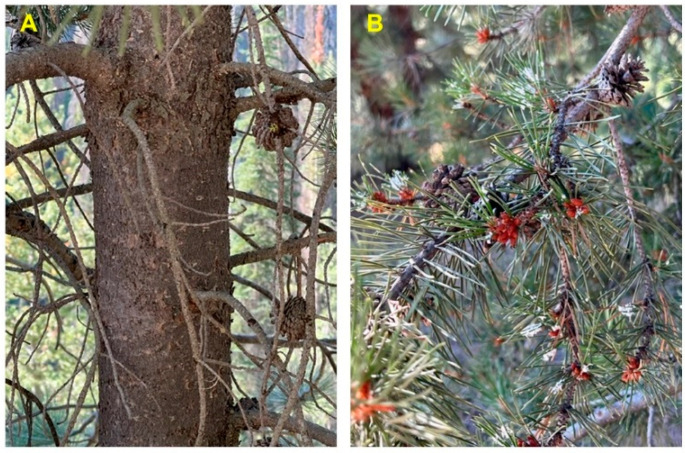
*Pinus contorta* subsp. *latifolia* from southern Idaho. (**A**): bark. (**B**): leaves and cones. Photographs by K. Swor.

*Pinus contorta* Douglas ex Loudon subsp. *latifolia* (Engelm. ex S. Watson) Critchf. (Rocky Mountain lodgepole pine, Pinaceae) is found in the Rocky Mountains of western North America, from the Yukon, south through Colorado (Figure 5). There are two other subspecies of *P. contorta*, *P. contorta* subsp. *contorta* Douglas ex Loudon (the shore pine), which ranges along the Pacific coast from southern Alaska, south to northwestern California, and *P. contorta* subsp. *murrayana* (Balf.) Engelm. (the Sierra lodgepole pine), which ranges in the Cascade Range in Washington and Oregon, south into northern California and the Sierra Nevada Range (Figure 5) [10,11]. The gray-brown bark of *P. contorta* subsp. *latifolia* is thin and scaly, while the needles are 4–8 cm long and in pairs (Figure 6).

**Figure 7:**
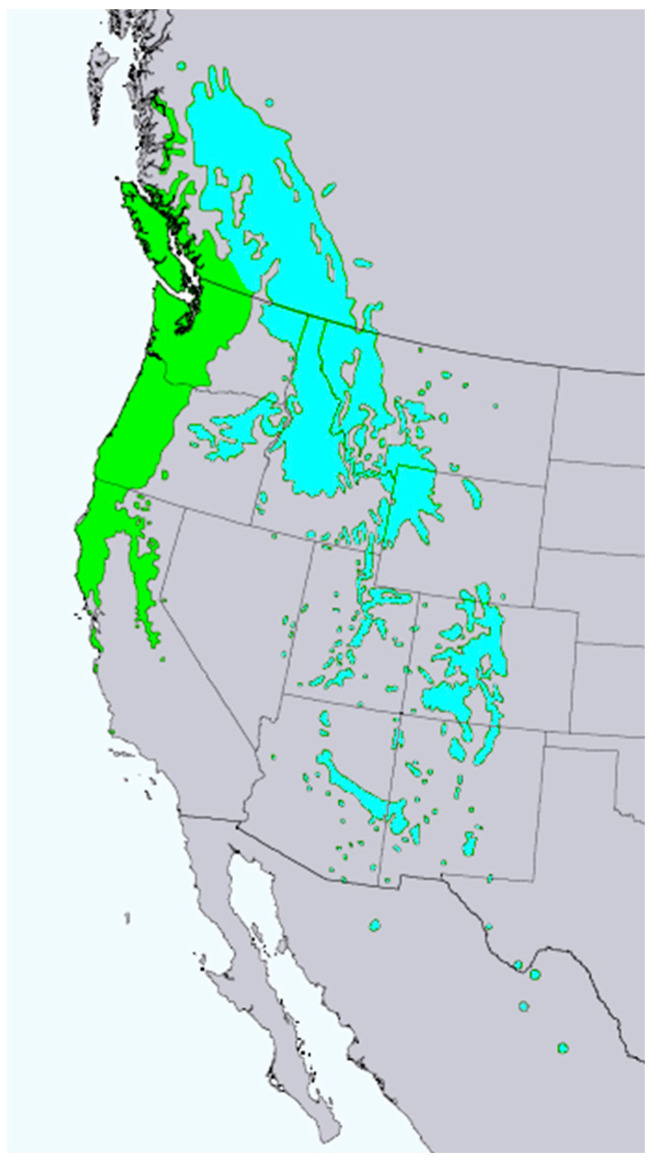
Natural range of *Pseudotsuga menziesii*. 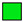 *P. menziesii* var. *menziesii*. 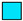 *P. menziesii* var. *glauca* [6].

**Figure 8:**
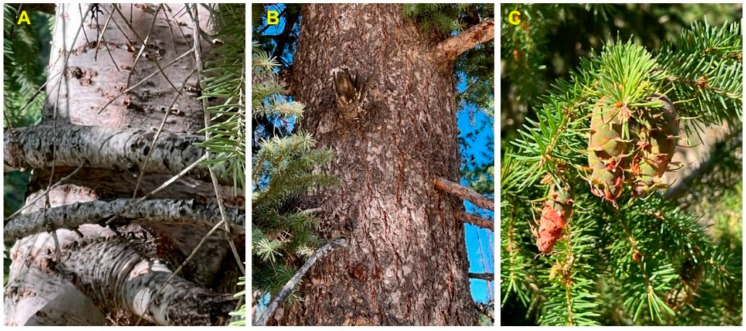
*Pseudotsuga menziesii* var. *glauca* from southern Idaho. (**A**): bark of young tree. (**B**): bark of old tree. (**C**): leaves and cones. Photographs by K. Swor.

*Pseudotsuga menziesii* (Mirb.) Franco (syn. *Abies menziesii* Mirb.) (Rocky Mountain Douglas fir, Pinaceae) is an important timber tree native to western North America [12]. The tree has been introduced to many temperate regions throughout the world. There are two varieties of Douglas fir, *P. menziesii* var. *menziesii* (coastal Douglas fir), which ranges from coastal British Columbia south through the Cascades into the Coastal and Sierra Nevada mountains of northern California, and *P. menziesii* var. *glauca* (Mayr) Franco (Rocky Mountain Douglas fir), which ranges from central British Columbia south into Arizona and New Mexico (Figure 7) [13]. There are populations of *P. menziesii* in Mexico that are morphologically similar to *P. menziesii* var. *glauca* that have been referred to as *Pseudotsuga menziesii* var. *oaxacana* Debreczy & I. Rácz [14], but there is little support for this particular taxon [15]. The bark on young trees is thin, smooth, gray, and covered with resin blisters. On mature trees, it is thicker (3–6 cm) and furrowed. The leaves are needles (2–3 cm long) spirally arranged around the branch (Figure 8).

The essential oils of both varieties (*menziesii* and *glauca*) have been extensively investigated by von Rudloff [16] and by Adams and co-workers [15]. The coastal Douglas fir has been characterized by relatively high concentrations of β-pinene (20–35%), terpinolene (5–20%), and terpinen-4-ol (5–15%), while the Rocky Mountain Douglas fir has shown large concentrations of camphene (20–30%), bornyl acetate (20–30%), and α-pinene (15–20%) [16]. In this work, the leaf essential oils from three individuals collected in southern Idaho have been obtained and the essential oil compositions determined using gas chromatographic methods. A comparison with Douglas fir essential oils from coastal, Rocky Mountain, and samples cultivated outside North America has also been carried out.

**Figure 9:**
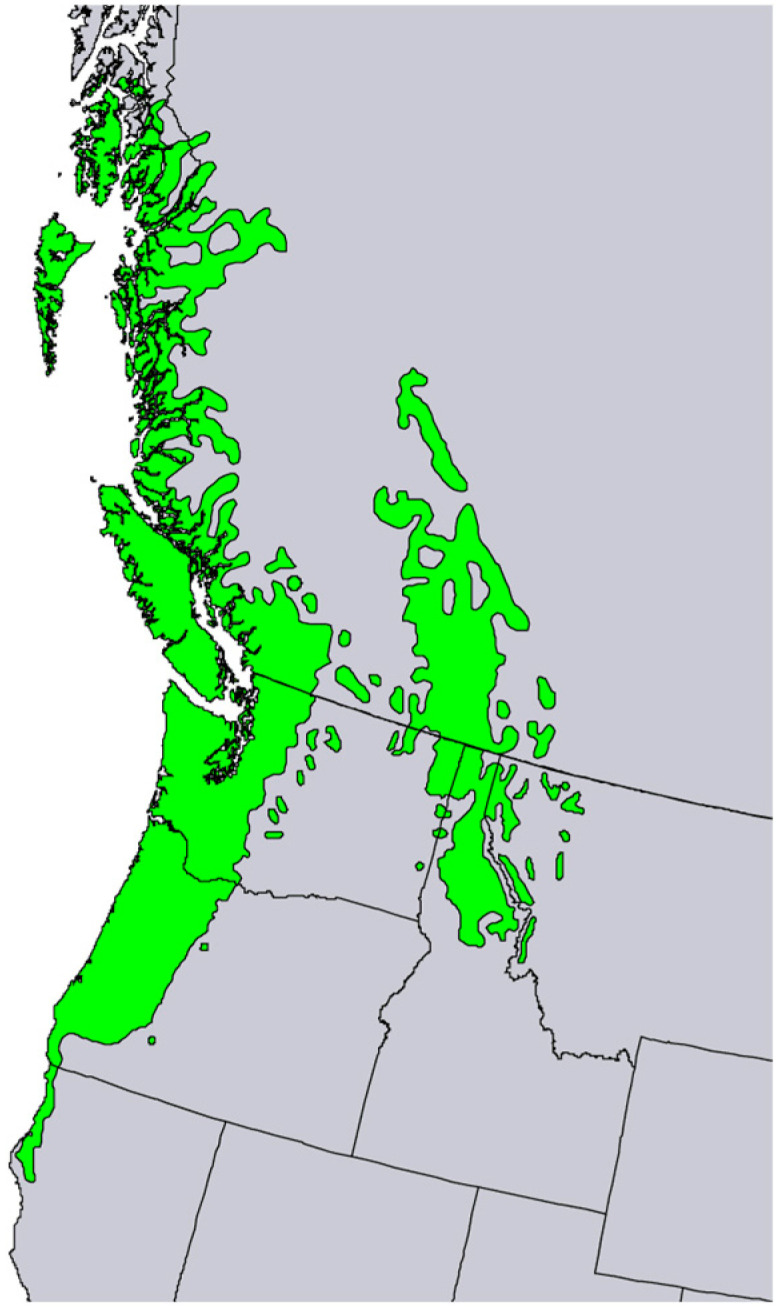
Native range of *Thuja plicata* [6].

**Figure 10:**
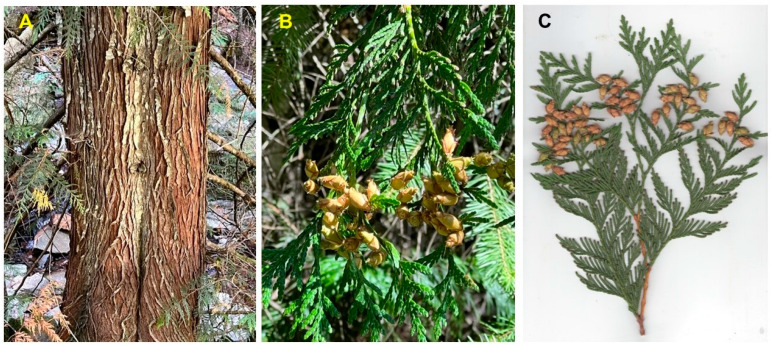
*Thuja plicata* from northern Idaho. (**A**): bark. (**B**): foliage and cones. (**C**): scan of foliage and cones. Photographs by K. Swor.

*Thuja plicata* Donn ex D. Don (western red cedar, Cupressaceae) is a large to very large evergreen tree native to western North America, ranging along the Cascade-Coastal Mountain Ranges from southeastern Alaska to northern California, and inland in the Rocky Mountains from British Columbia to the panhandle of northern Idaho (Figure 9) [17]. Western red cedar is an important timber-producing tree and has been introduced to other temperate zone locations, including Europe, Great Britain, Australia, and New Zealand [18,19,20,21,22,23,24,25]. The thin, gray-brown bark forms vertical bands of fissures; the branch termini form flat boughs with scale-like leaves; the cones are 10–18 mm long and 4–5 mm wide with overlapping scales (Figure 10). The Nez Perce people used an infusion of the foliage to treat colds and coughs [5].

## 2. Results and Discussion

### 2.1. Essential Oil Composition

Essential oils of the conifer species were obtained by hydrodistillation and the essential oil compositions determined using gas chromatography (GC-MS and GC-FID).

#### 2.1.1. *Abies lasiocarpa* var. *lasiocarpa*

The foliage (branch tips and leaves, no cones) from two individual mature *A. lasiocarpa* var. *lasiocarpa* trees (*A.l.l.* #1 and *A.l.l.* #2) from southern Idaho were hydrodistilled to give colorless essential oils in 1.611% and 1.857% yield based on masses of fresh/frozen plant material). Gas chromatographic analysis of the essential oils was carried out to assess the chemical compositions (Table 1).

The major components in *A. lasiocarpa* essential oils were limonene (20.3% and 34.6%), bornyl acetate (24.7% and 18.5%), β-pinene (13.6% and 9.3%), camphene (10.9% and 7.4%), and α-pinene (5.0% and 4.5%). The compositions are consistent with those reported by Adams and co-authors for Rocky Mountain subalpine fir from Montana and Utah [2].

#### 2.1.2. *Picea engelmannii* subsp. *engelmannii*

Hydrodistillation of the branch tips and leaves of *P. engelmannii* subsp. *engelmannii* (*P.e.e.*) gave a yellow essential oil in 0.912% yield based on mass of fresh/frozen plant material. The essential oil composition is listed in Table 2. The essential oil was rich in oxygenated monoterpenoids (50.2%), including camphor (22.8%), borneol (8.3%), and camphene hydrate (6.0%), as well as monoterpene hydrocarbons, (38.2%) myrcene (11.7%) and camphene (6.0%). There have been previous examinations of *P. engelmannii* from Arizona [31] and from Poland [32].

**Figure 11:**
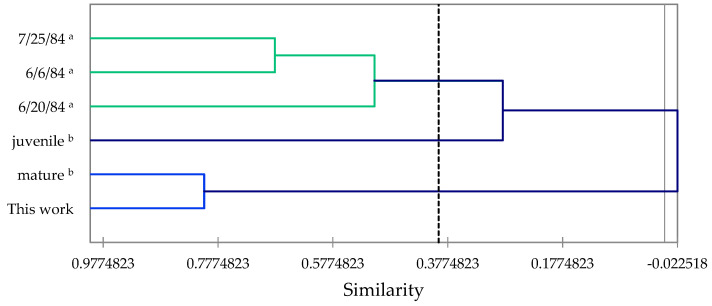
Dendrogram based on hierarchical cluster analysis of *Picea engelmannii* foliar essential oil compositions. ^a^ Wagner et al., 1989 [31]. ^b^ Mardarowicz et al., 2004 [32].

An agglomerative hierarchical cluster (AHC) analysis was carried out to reveal the similarities between these essential oil samples (Figure 11). The samples from Arizona (sampled on 6 June, 20 June, and 25 July of 1984) showed wide variation in essential oil composition (<70% similarity) for the three dates. Mardarowicz and co-workers sampled a mature tree and saplings of cultivated trees in Poland [32]. The juvenile and mature foliar essential oils were very different in composition, but the composition of the mature foliar essential oil is similar (>80% similarity) to the *P. engelmannii* essential oil from Idaho. Thus, for example, the major components in the mature foliar essential oil from Poland were camphor (14.9%), borneol (5.2%), camphene hydrate (5.0%), myrcene (12.2%), and camphene (3.5%). Interestingly, the Poland sample had 5.6% benzaldehyde, which was not observed in the Idaho sample.

#### 2.1.3. *Pinus contorta* subsp. *latifolia*

Leaves (needles) of *P. contorta* subsp. *latifolia* from two mature trees (*P.c.l.* #1 and *P.c.l.* #2) were hydrodistilled to give colorless essential oils in 3.105% and 1.702% yield based on masses of fresh/frozen plant material. The gas chromatographic results are summarized in Table 3. The major components in the essential oils were β-pinene (27.0% and 20.3%), β-phellandrene (21.8% and 20.9%), δ-3-carene (3.6% and 11.0%), (2*E*)-hexenal (7.1% and 5.3%), α-pinene (5.0% and 4.0%), and α-terpineol (6.7% and 5.7%).

**Figure 12:**
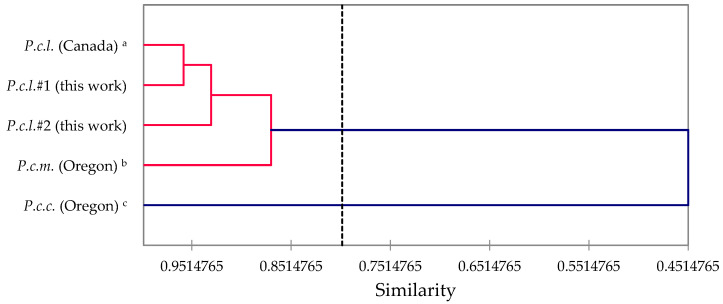
Dendrogram based on hierarchical cluster analysis of *Pinus contorta* leaf essential oil compositions. *P.c.l.* = *Pinus contorta* subsp. *latifolia*, *P.c.m.* = *Pinus contorta* subsp. *murrayana*, *P.c.c.* = *Pinus contorta* subsp. *contorta*. ^a^ Pauly and von Rudloff, 1971 [33]. ^b^ Ankney et al., 2021 [10]. ^c^ Ankney et al., 2022 [11].

In order to compare and contrast the essential oil compositions of *P. contorta* subsp. *latifolia* from Idaho with *P. contorta* subsp. *latifolia* from Alberta, Canada [33], *P. contorta* subsp. *murrayana* from Oregon [10], and *P. contorta* subsp. *contorta* from Oregon [11], an AHC analysis was carried out (Figure 12). The three *P. contorta* subsp. *latifolia* samples show > 90% similarity, while *P. contorta* subsp. *murrayana* shows 87% similarity to the *latifolia* subspecies. The least similar in essential oil composition is *P. contorta* subsp. *contorta* with only 45% similarity. Although β-phellandrene was the major component in all of the *P. contorta* essential oils, β-pinene was only a minor component (0.5%) in *P. contorta* subsp. *contorta*, but terpinen-4-ol was a major component (11.0%) in *P. contorta* subsp. *contorta*, which account for the lack of similarity of this essential oil.

#### 2.1.4. *Pseudotsuga menziesii* var. *glauca*

Hydrodistillation of the leaves (needles) of *P. menziesii* from three individual trees (*P.m.g.* #1, *P.m.g.* #2, and *P.m.g.* #3) from southern Idaho gave pale-yellow essential oils in 0.658–1.462% yield based on masses of fresh/frozen plant material. The chemical compositions of the three *P. menziesii* samples are compiled in Table 4.

**Figure 13:**
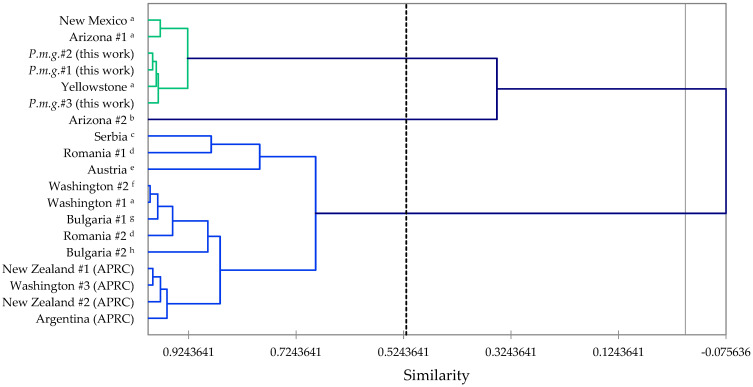
Dendrogram based on hierarchical cluster analysis of *Pseudotsuga menziesii* chemical compositions. *P.m.g.* = *Pseudotsuga menziesii* var. *glauca*. APRC = Commercial essential oil samples from the Aromatic Plant Research Center collection. ^a^ Von Rudloff, 1973 [16]. ^b^ Wagner et al., 1989 [31]. ^c^ Mitić et al., 2021 [39]. ^d^ Pădure et al., 2008 [38]. ^e^ Buchbauer et al., 1994 [35]. ^f^ Adams, 2012 [34]. ^g^ Jirovetz et al., 2000 [36]. ^h^ Jirovetz et al., 2000 [37].

**Figure 14:**
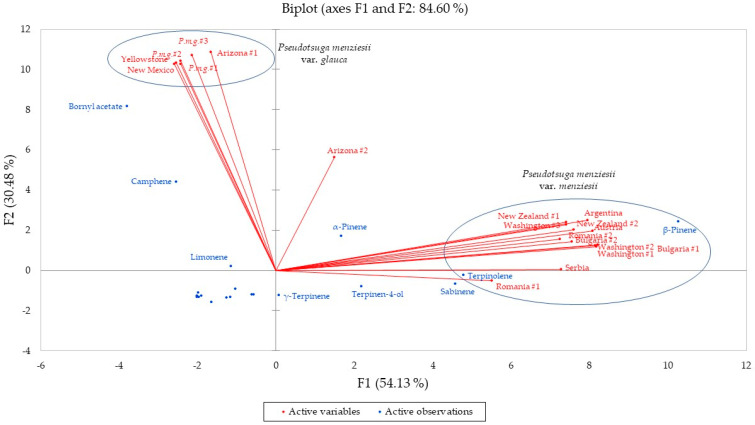
Biplot based on principal component analysis of *Pseudotsuga menziesii* chemical compositions. *P.m.g.* = *Pseudotsuga menziesii* var. *glauca*. APRC = Commercial essential oil samples from the Aromatic Plant Research Center collection.

The major components in the essential oils were bornyl acetate (38.7–41.1%), camphene (15.0–19.5%), α-pinene (6.3–11.2%), and limonene (3.9–5.4%), confirming the identification of these samples as Rocky Mountain Douglas fir (*P. menziesii* var. *glauca*) [15,16]. In order to complement the volatile phytochemical differences between *P. menziesii* var. *menziesii* [15,34] and *P. menziesii* var. *glauca* as well as place samples from outside North America [31,35,36,37,38,39] into chemical context, both agglomerative hierarchical cluster (AHC) analysis (Figure 13) and principal component analysis (PCA, Figure 14) were carried out using the percent compositions of the major components (Supplementary Table S1).

There are two well-defined clusters based on the AHC. Cluster 1 is a cluster made up of samples from Idaho (this work), Yellowstone, Arizona, and New Mexico; dominated by bornyl acetate and camphene; and is clearly *P. menziesii* var. *glauca* based on the volatile phytochemicals and the geographical locations. Cluster 2 is made up of samples from Washington state (*P. menziesii* var. *menziesii*) as well as cultivated samples from Serbia, Romania, Austria, Bulgaria, Argentina, and New Zealand, and is defined by large concentrations of β-pinene, terpinolene, and sabinene. The chemical compositions of the non-North American cultivated samples are consistent with the *menziesii* variety and are likely derived from *P. menziesii* var. *menziesii* parents. There is one sample from Arizona [31] that does not fit into either the *glauca* or the *menziesii* varieties, and likely represents an “Interior Intermediate” chemotype [16].

The PCA verifies the AHC with the *P. menziesii* var. *glauca* group positively correlating with bornyl acetate and camphene. The *P. menziesii* var. *menziesii* group, on the other hand, positively correlates with β-pinene, terpinolene, and sabinene. The “Interior Intermediate” sample from Arizona correlates most strongly with camphene, α-pinene, β-pinene, and limonene.

#### 2.1.5. *Thuja plicata*

Hydrodistillation of *T. plicata* foliage from five different trees (*T.p.* #1–*T.p.* #5) growing near Coeur d’Alene, Idaho, gave pale-yellow essential oils in yields ranging from 0.99% to 4.70% based on masses of fresh/frozen plant material. The essential oils were analyzed by gas chromatographic methods (GC-MS and GC-FID, Table 5).

The essential oils were dominated by α-thujone (72.5–77.8%) and β-thujone (5.2–8.2%), with notable quantities of sabinene (1.4–3.0%) and terpinene-4-ol (2.2–3.1%). The compositions observed are very similar to those previously reported by von Rudloff et al. (both coastal and interior populations of western North America) [40], Nikolić et al. (Serbia) [25], Tsiri et al. (Poland) [23], and Lis et al. (Poland) [24]. That is, the foliar essential oils of *T. plicata*, regardless of geographical location, have been dominated by α-thujone, with lesser amounts of β-thujone, sabinene, and terpinen-4-ol [40]. Samples from Poland, however, showed relatively high concentrations of fenchone (7.1–11.3%), which were not reported in the samples from Serbia or from Idaho. *Thuja plicata* has shown low genetic diversity [41], which is consistent with the low variation in essential oil composition.

The foliar essential oil of *T. plicata* has shown insecticidal [42], insect antifeedant [25], as well as antibacterial and antifungal [25,43] activities. The biological activities of *T. plicata* essential oil can be attributed to the major component, α-thujone. The toxicity of α-thujone has been determined to be due to modulation of the γ-aminobutyric acid (GABA) type A receptor [44]. α-Thujone, and to a lesser extent, β-thujone have shown antinociceptive activities in a rodent model [45]. In addition, thujone has shown anti-inflammatory activity due to inhibition of induced interleukin (IL-6 and IL-8) release [46]. Thus, the biological properties of α-thujone are consistent with the Native American herbal medicinal uses of the plant.

A comparison of essential oil compositions between the five species of conifers in this study (see Supplementary Table S2) shows that *A. lasiocarpa* var. *lasiocarpa* and *P. menziesii* var. *glauca* have similar compositions, both species are rich in bornyl acetate, camphene, and limonene. On the other hand, *P. engelmanii* var. *engelmanii* (dominated by camphor and myrcene), *Pinus contorta* subsp. *latifolia* (rich in β-pinene and β-phellandrene), and *Thuja plicata* (dominated by thujones), are completely dissimilar in composition with all of the other species.

### 2.2. Terpenoid Enantiomeric Distributions

Chiral gas chromatographic–mass spectral analyses were carried out on the essential oils of *Abies lasiocarpa* var. *lasiocarpa*, *Picea engelmannii* subsp. *engelmannii*, *Pinus contorta* subsp. *latifolia*, *Pseudotsuga menziesii* var. *glauca*, and *Thuja plicata* to discern the enantiomeric distribution of chiral monoterpenoids (see Table 6). Interestingly, the (−)-enantiomers were the predominant stereoisomers for α-pinene, camphene, sabinene, β-pinene, limonene, β-phellandrene, linalool, terpinen-4-ol, borneol, and α-terpineol for essential oils of the Pinaceae. In contrast, the (+)-enantiomers of α-thujene, α-pinene, sabinene, β-pinene, limonene, *cis*-sabinene hydrate, β-thujone, terpinen-4-ol, and α-terpineol were dominant in *T. plicata* (Cupressaceae) essential oils.

Consistent with these findings, the (−)-enantiomers predominate for camphene, β-pinene, limonene, β-phellandrene, and α-terpineol in the Pinaceae essential oils of *Abies concolor*, *Abies balsamea* [47], *Picea pungens* [48], *Pinus ponderosa*, *Pinus contorta*, and *Pinus flexilis* [11]. Likewise, while (+)-α-thujene was the exclusive enantiomer in *T. plicata*, (−)-α-thujene was dominant in *A. concolor* and *A. balsamea* [47]. Furthermore, in the wood essential oils of *Sequoia sempervirens* (Cupressaceae), (+)-α-pinene, (+)-limonene, and (+)-α-terpineol predominated [49]. In *Juniperus* (Cupressaceae) essential oils from southwestern Idaho, (+)-α-thujene, (+)-α-pinene, (+)-limonene, and (+)-cis-sabinene hydrate predominated [50].

## 3. Materials and Methods

### 3.1. Plant Material

Samples of *A. lasiocarpa* var. *lasiocarpa*, *P. engelmannii* subsp. *engelmannii*, *P. contorta* subsp. *latifolia*, and *P. menziesii* var *glauca* were collected from individual trees near Featherville, Boise National Forest, Idaho, on 25 August 2022 (Table 7). Several subsamples were collected from each individual tree. Voucher specimens (*A. lasiocarpa* var. *lasiocarpa*, WNS-All-5856; *P. engelmannii* subsp. *engelmannii*, WNS-Pee-5881; *P. contorta* subsp. *latifolia*, WNS-Pcl-5852; and *P. menziesii* var *glauca*, WNS-Pmg-5845) have been deposited in the University of Alabama in Huntsville herbarium. The trees were identified in the field by K. Swor and W.N. Setzer and later verified by comparison with samples from the New York Botanical Garden Virtual Herbarium (https://sweetgum.nybg.org/science/vh/, accessed on 26 September 2022). The samples were freshly frozen (−20 °C) until distilled. The foliage from each individual was hydrodistilled for 4 h using a Likens-Nickerson apparatus to give the essential oils (Table 7). The foliage of *T. plicata* was collected from several trees near Coeur d’Alene, Idaho on 21 September 2022. A voucher specimen of *T. plicata* (WNS-Tp-6050) has been deposited in the University of Alabama in Huntsville herbarium. The fresh foliage was stored frozen (−20 °C) until distilled. The *T. plicata* foliage from each tree was hydrodistilled using a Likens-Nickerson apparatus for 4 h to give pale-yellow essential oils with pungent odors (see Table 7).

### 3.2. Gas Chromatographic Analyses

Gas chromatography–mass spectrometry (GC-MS) was carried out using the instrumentation and conditions previously reported [51]: Shimadzu GC-MS-QP2010 Ultra (Shimadzu Scientific Instruments, Columbia, MD, USA), ZB-5ms GC column (5% phenyl polydimethylsiloxane, 60 m × 0.25 mm × 0.25 μm film thickness) (Phenomenex, Torrance, CA, USA), injector and detector temperatures = 260 °C, helium carrier gas (column head pressure = 208.5 kPa, flow rate = 2.00 mL/min), GC oven temperature program = 50 °C start, ramp to 260 °C at 2 °C/min. For each essential oil sample, 1.0 μL of a 5% (*w*/*v*) solution in CH_2_Cl_2_ was injected (splitting mode of 24.5:1). Retention index (RI) values were determined using a homologous series of *n*-alkanes [26]. The essential oil compositions were ascertained by comparison of their RI values and MS fragmentation patterns with those reported in the databases [27,28,29,30] using the LabSolutions GCMS solution software version 4.45 (Shimadzu Scientific Instruments, Columbia, MD, USA).

Gas chromatography with flame-ionization detection (GC-FID) was carried out as previously reported [51]: Shimadzu GC 2010 instrument with FID detector (Shimadzu Scientific Instruments, Columbia, MD, USA), ZB-5 GC column (60 m × 0.25 mm × 0.25 μm film thickness) (Phenomenex, Torrance, CA, USA), using the same operating conditions as above for GC-MS. The percent compositions were determined from raw peak areas without standardization.

Chiral GC-MS was carried out as previously reported [51]: Shimadzu GC-MS-QP2010S instrument (Shimadzu Scientific Instruments), Restek B-Dex 325 column (30 m × 0.25 mm diameter × 0.25 μm film thickness) (Restek Corp., Bellefonte, PA, USA), injector and detector temperatures = 240 °C. Helium carrier gas (column head pressure = 53.6 kPa, flow rate of 2.00 mL/min), GC oven program = 50 °C start, hold for 5 min, increased to 100 °C at 1.0 °C/min, then increased to 220 °C at 2 °C/min. For each essential oil sample, 0.3 μL of a 5% (*w*/*v*) solution in CH_2_Cl_2_ was injected (splitting mode = 24.0:1). The enantiomers were determined by comparison of retention times with authentic samples obtained from Sigma-Aldrich (Milwaukee, WI, USA). The enantiomer percentages were determined from raw peak areas.

### 3.3. Multivariate Analyses

For the agglomerative hierarchical cluster (AHC) analyses, the essential oil compositions for each species were treated as operational taxonomic units (OTUs), and the percentages of the most abundant essential oil components were used to delineate the chemical associations between the essential oil samples (*P. engelmannii*: tricyclene, α-pinene, camphene, benzaldehyde, β-pinene, myrcene, δ-3-carene, limonene, β-phellandrene, 1,8-cineole, fenchone, linalool, camphor, camphene hydrate, borneol, terpinen-4-ol, α-terpineol, piperitone, bornyl acetate, longifolene, (*E*)-β-caryophyllene, and α-cadinol; *Pinus contorta*: (2*E*)-hexenal, α-pinene, β-pinene, myrcene, δ-3-carene, 1,4-cineole, α-terpinene, limonene, β-phellandrene, γ-terpinene, terpinolene, terpinen-4-ol, α-terpineol, chavicol, thymol; *Pseudotsuga menziesii*: santene, tricyclene, α-pinene, camphene, sabinene, β-pinene, δ-3-carene, limonene, β-phellandrene, (*Z*)-β-ocimene, (*E*)-β-ocimene, γ-terpinene, terpinolene, camphene hydrate, borneol, terpinen-4-ol, α-terpineol, bornyl acetate, citronellyl acetate, geranyl acetate). Pearson correlation was used to measure similarity, and the unweighted pair group method with arithmetic average (UPGMA) was used for cluster definition. Principal component analysis (PCA) was performed for the visual verification of the essential oil inter-relationships of the different infraspecific taxa of *P. menziesii* using the major components as variables with a Pearson correlation matrix. The AHC and PCA analyses were performed using XLSTAT v. 2018.1.1.62926 (Addinsoft, Paris, France).

## 4. Conclusions

The essential oils of Rocky Mountain subalpine fir (*Abies lasiocarpa* var. *lasiocarpa*) (Pinaceae), Engelmann spruce (*Picea engelmannii* subsp. *engelmannii*) (Pinaceae), Rocky Mountain lodgepole pine (*Pinus contorta* subsp. *latifolia*) (Pinaceae), Rocky Mountain Douglas fir (*Pseudotsuga menziesii* var. *glauca*) (Pinaceae), and Western red cedar (*Thuja plicata*) (Cupressaceae) from Idaho have been obtained and analyzed by gas chromatographic methods. The essential oil compositions obtained in this work are qualitatively similar, but quantitatively different, to previously reported compositions and confirm and complement the previous reports. The quantitative similarities or differences in essential oil compositions are important; any commercial, cosmetic, fragrance, or medicinal uses of the essential oils derived from these plant species may depend on differences due to geographical, edaphic, climatic, or genetic differences. As far as we are aware, this report presents the first comprehensive analysis of the chiral terpenoid components in *Abies lasiocarpa*, *Picea engelmannii*, *Pinus contorta*, *Pseudotsuga menziesii*, and *Thuja plicata*. The (−)-enantiomers seem to predominate for many monoterpenoid constituents in the Pinaceae, but the (+)-enantiomers are favored in the Cupressaceae. Nevertheless, additional research on essential oils of the Pinaceae and Cupressaceae is needed (e.g., higher sampling variability and different geographical locations) to describe the chemical profiles, chemical compositions and enantiomeric distributions more reliably in the various species and infraspecific taxa of these two families.

## Figures and Tables

**Table 1:** Chemical compositions (percent) of the foliar essential oils of *Abies lasiocarpa* var. *lasiocarpa* (Rocky Mountain subalpine fir) from southern Idaho.

RI_calc_	RI_db_	Compound	*A.l.l. *#1	*A.l.l. *#2
881	880	Santene	1.2	1.5
923	923	Tricyclene	1.1	0.7
926	925	α-Thujene	0.1	0.1
934	933	α-Pinene	5.0	4.5
950	950	Camphene	10.9	7.4
966	969	Methyl 2-methyl-3-hexenoate	tr	tr
972	972	Sabinene	tr	0.1
978	978	β-Pinene	13.6	9.3
989	989	Myrcene	1.1	1.5
1005	1004	*p*-Mentha-1(7),8-diene	tr	tr
1007	1007	α-Phellandrene	0.2	0.3
1010	1009	δ-3-Carene	tr	0.3
1017	1017	α-Terpinene	0.1	0.1
1025	1025	*p*-Cymene	0.1	0.2
1031	1030	Limonene	20.3	34.6
1035	1031	β-Phellandrene	6.7	7.1
1036	1034	(*Z*)-β-Ocimene	---	0.1
1038	1041	2-Heptyl acetate	0.2	---
1046	1046	(*E*)-β-Ocimene	---	0.7
1057	1057	γ-Terpinene	0.2	0.1
1069	1069	*cis*-Sabinene hydrate	tr	tr
1086	1086	Terpinolene	0.7	0.4
1089	1090	Fenchone	0.1	tr
1091	1093	*p*-Cymenene	tr	0.1
1101	1101	Linalool	0.5	0.4
1107	1108	Maltol	tr	---
1113	1113	(*E*)-4,8-Dimethylnona-1,3,7-triene	tr	tr
1118	1119	*endo*-Fenchol	tr	tr
1125	1124	*cis-p*-Menth-2-en-1-ol	0.4	0.3
1127	1126	α-Campholenal	tr	---
1142	1142	*trans-p*-Menth-2-en-1-ol	0.3	0.2
1147	1145	Camphor	0.2	tr
1151	1151	Citronellal	tr	tr
1155	1156	Camphene hydrate	0.1	tr
1158	1157	*iso*-Isopulegol	tr	tr
1164	1165	*iso*-Borneol	tr	---
1172	1173	Borneol	0.2	0.2
1179	1179	2-Isopropenyl-5-methyl-4-hexenal	0.1	0.2
1180	1180	Terpinen-4-ol	0.2	0.2
1187	1187	Cryptone	tr	tr
1188	1188	*p*-Cymen-8-ol	tr	tr
1191	1192	Methyl salicylate	0.1	tr
1195	1195	α-Terpineol	0.4	0.3
1197	1196	*cis*-Piperitol	tr	0.1
1209	1208	*trans*-Piperitol	0.2	0.1
1217	1217	*endo*-Fenchyl acetate	tr	0.1
1228	1227	Citronellol	0.2	tr
1229	1229	Thymyl methyl ether	3.5	tr
1232	1231	*trans*-Chrysanthyl acetate	tr	tr
1250	1252	Isopentyl hexanoate	tr	---
1251	1255	Geraniol	tr	---
1254	1254	Piperitone	2.8	3.0
1257	1257	Methyl citronellate	tr	tr
1286	1285	Bornyl acetate	24.7	18.5
1288	1287	*iso*-Bornyl acetate	0.2	tr
1291	1289	Thymol	tr	1.5
1292	1293	2-Undecanone	tr	tr
1314	1314	Carvenolide	0.1	0.1
1334	1335	*cis*-Piperityl acetate	0.1	tr
1350	1350	Citronellyl acetate	1.0	0.7
1358	1361	Neryl acetate	tr	0.1
1378	1378	Geranyl acetate	0.4	0.6
1390	1390	*trans*-β-Elemene	tr	tr
1409	1408	Acora-3,7(14)-diene	---	tr
1410	1411	Longifolene	tr	0.1
1418	1414	α-Cedrene	---	0.1
1451	1452	α-Himachalene	tr	0.1
1452	1452	(*E*)-β-Farnesene	tr	tr
1465	1465	Bornyl butyrate	tr	tr
1474	1475	Selina-4,11-diene	tr	tr
1482	1483	Citronellol isobutyrate	---	tr
1489	1489	β-Selinene	0.1	0.3
1495	1494	δ-Decalactone	0.3	---
1496	1494	α-Selinene	0.1	0.5
1504	1504	(*E*,*E*)-α-Farnesene	tr	tr
1508	1508	β-Bisabolene	0.3	0.9
1511	1511	(*Z*)-γ-Bisabolene	0.1	0.1
1526	1525	Citronellyl butyrate	0.1	0.1
1541	1541	(*E*)-α-Bisabolene	tr	0.1
1555	1555	Geranyl butyrate	0.1	0.1
1560	1560	(*E*)-Nerolidol	0.3	0.1
1567	1564	Citronellyl 2-methylbutanoate	0.1	0.1
1572	1572	Citronellyl isovalerate	tr	tr
1596	1596	Geranyl 2-methylbutanoate	0.1	tr
1603	1604	Geranyl isovalerate	tr	tr
1685	1686	*epi*-α-Bisabolol	tr	tr
1688	1688	α-Bisabolol	0.8	1.8
1715	1716	Citronellyl hexanoate	tr	tr
1747	1748	Geranyl hexanoate	tr	tr
1831	1832	(2*Z*,6*E*)-Farnesyl acetate	tr	tr
1990	1989	Manoyl oxide	tr	tr
2050	2049	Abietatriene	tr	tr
2084	2086	Abietadiene	tr	tr
2143	2147	Abienol	tr	0.1
		Monoterpene hydrocarbons	61.2	69.1
		Oxygenated monoterpenoids	35.8	26.8
		Sesquiterpene hydrocarbons	0.5	2.0
		Oxygenated sesquiterpenoids	1.1	1.9
		Diterpenoids	traces	0.2
		Benzenoid aromatics	0.1	traces
		Others	0.4	traces
		Total identified	99.2	100.0

RI_calc_ = Retention index values determined using the method of van den Dool and Kratz [26]. RI_db_ = Reference retention index values from the databases [27,28,29,30]. *A.l.l.* = *Abies lasiocarpa* var. *lasiocarpa*. tr = trace (<0.05%).

**Table 2:** Chemical composition (percent) of the foliar essential oil of *Picea engelmannii* subsp. *engelmannii* from southern Idaho.

RI_calc_	RI_db_	Compound	%
777	769	(2*Z*)-Penten-1-ol	0.1
780	772	Prenol	0.1
797	797	(3*Z*)-Hexenal	tr
803	801	Hexanal	tr
849	849	(2*E*)-Hexenal	0.4
851	853	(3*Z*)-Hexenol	0.2
865	860	1-Hexanol	tr
881	880	Santene	0.2
923	923	Tricyclene	0.4
926	925	α-Thujene	0.1
934	932	α-Pinene	3.6
948	948	α-Fenchene	tr
950	950	Camphene	6.0
972	971	Sabinene	0.3
978	978	β-Pinene	2.4
990	989	Myrcene	11.7
1008	1006	α-Phellandrene	0.1
1010	1008	δ-3-Carene	3.7
1017	1017	α-Terpinene	0.1
1025	1024	*p*-Cymene	0.2
1030	1030	Limonene	4.4
1032	1031	β-Phellandrene	4.3
1033	1032	1,8-Cineole	2.4
1035	1034	(*Z*)-β-Ocimene	tr
1045	1045	(*E*)-β-Ocimene	tr
1055	1056	Isoamyl butyrate	tr
1057	1057	γ-Terpinene	0.2
1063	1064	3-Methyl-2-butenyl butyrate	0.1
1071	1069	*cis*-Linalool oxide (furanoid)	0.1
1081	1082	*p*-Mentha-2,4(8)-diene	tr
1085	1086	Terpinolene	0.7
1086	1086	*trans*-Linalool oxide (furanoid)	0.1
1088	1090	Fenchone	0.2
1090	1093	*p*-Cymenene	0.1
1094	1094	Methyl benzoate	0.1
1101	1101	Linalool	1.2
1121	1123	*endo*-Fenchol	0.1
1126	1124	*cis-p*-Menth-2-en-1-ol	0.2
1149	1145	Camphor	22.8
1153	1151	Citronellal	tr
1156	1156	Camphene hydrate	6.0
1163	1165	Isoborneol	0.2
1174	1173	Borneol	8.3
1179	1179	2-Isopropenyl-5-methyl-4-hexenal	0.1
1181	1180	Terpinen-4-ol	0.6
1187	1186	*p*-Cymen-8-ol	0.5
1196	1195	α-Terpineol	2.8
1198	1197	Estragole (=Methyl chavicol)	0.1
1207	1205	Verbenone	0.1
1219	1218	*trans*-Carveol	0.1
1227	1227	Citronellol	0.7
1229	1229	Thymyl methyl ether	0.2
1250	1249	Geraniol	0.1
1254	1254	Piperitone	0.8
1284	1285	Bornyl acetate	2.4
1312	1314	Carvenolide	0.1
1348	1348	α-Longipinene	0.3
1372	1372	Longicyclene	0.1
1377	1378	Geranyl acetate	0.2
1390	1390	*trans*-β-Elemene	0.1
1409	1411	Longifolene	0.8
1419	1417	(*E*)-β-Caryophyllene	0.1
1438	1439	Isoamyl benzoate	0.1
1445	1443	Prenyl benzoate	0.1
1453	1452	(*E*)-β-Farnesene	0.1
1488	1487	β-Selinene	tr
1491	1490	γ-Amorphene	tr
1495	1497	α-Selinene	0.1
1498	1497	α-Muurolene	0.1
1507	1508	β-Bisabolene	tr
1509	1511	β-Curcumene	tr
1510	1511	(Z)-γ-Bisabolene	tr
1510	1512	γ-Cadinene	0.2
1518	1518	δ-Cadinene	0.5
1526	1528	(*E*)-γ-Bisabolene	0.1
1536	1538	α-Cadinene	tr
1540	1541	(*E*)-α-Bisabolene	0.1
1561	1561	(*E*)-Nerolidol	0.1
1575	1575	Germacra-1(10),5-dien-4β-ol	0.3
1601	1600	α-Oplopenone	0.1
1613	1616	1,10-di-*epi*-Cubenol	tr
1626	1628	1-*epi*-Cubenol	0.1
1641	1640	τ-Cadinol	0.4
1643	1644	τ-Muurolol	0.4
1645	1643	α-Muurolol (=δ-Cadinol)	0.1
1655	1655	α-Cadinol	1.2
1657	1660	*neo*-Intermedeol	0.1
1686	1686	*epi*-α-Bisabolol	0.2
1731	1735	Oplopanone	0.5
1927	1934	Cembrene	0.6
1939	1931	Musk ambrette ^a^	0.1
1941	1947	(3*E*)-Cembrene A	0.2
1952	1947	α-Springene	0.1
1957	1961	(3*Z*)-Cembrene A	0.1
1997	1994	Manoyl oxide	0.1
2001	2000	9β-Isopimara-7,15-diene	0.1
2046	2038	Thunbergol A	1.0
2056	2058	Abietatriene	0.1
2088	2086	Abietadiene	tr
2149	2147	*cis*-Abienol	0.3
2233	2245	Palustral	0.8
2265	2266	Dehydroabietal	0.2
2295	2297	Methyl isopimarate	tr
2299	2302	Methyl levopimarate	tr
2300	2300	Tricosane	0.1
2309	2312	Abietal	tr
2400	2400	Tetracosane	tr
		Monoterpene hydrocarbons	38.2
		Oxygenated monoterpenoids	50.2
		Sesquiterpene hydrocarbons	2.5
		Oxygenated sesquiterpenoids	3.3
		Diterpenoids	3.4
		Benzenoid aromatics	0.4
		Others	1.0
		Total identified	99.1

RI_calc_ = Retention index values determined using the method of van den Dool and Kratz [26]. RI_db_ = Reference retention index values from the databases [27,28,29,30]. tr = trace (<0.05%). ^a^ May be a contaminant.

**Table 3:** Leaf essential oil compositions (percent) of *Pinus contorta* subsp. *latifolia* from southern Idaho.

RI_calc_	RI_db_	Compound	*P.c.l. *#1	*P.c.l. *#2
782	782	Prenol	0.1	0.1
797	797	(3*Z*)-Hexenal	1.0	0.7
799	801	Hexanal	1.4	1.0
826	828	2-Furfural	---	0.1
845	849	(2*E*)-Hexenal	7.1	5.3
847	853	(3*Z*)-Hexenol	0.5	0.5
923	923	Tricyclene	0.1	0.1
926	927	α-Thujene	0.1	0.1
933	932	α-Pinene	5.0	4.0
947	948	α-Fenchene	tr	0.1
949	950	Camphene	0.4	0.4
970	970	3,7,7-Trimethylcyclohepta-1,3,5-triene	tr	0.1
972	971	Sabinene	0.3	0.4
978	978	β-Pinene	27.0	20.3
989	989	Myrcene	4.2	3.0
1007	1006	α-Phellandrene	0.9	0.7
1008	1008	δ-3-Carene	3.6	11.0
1015	1015	1,4-Cineole	0.1	0.2
1017	1017	α-Terpinene	0.4	0.5
1019	1022	*m*-Cymene	---	tr
1024	1024	*p*-Cymene	0.2	0.5
1029	1030	Limonene	3.3	3.7
1031	1031	β-Phellandrene	21.8	20.9
1035	1034	(*Z*)-β-Ocimene	2.1	3.2
1045	1045	(*E*)-β-Ocimene	0.1	0.6
1057	1057	γ-Terpinene	0.4	0.7
1070	1069	*cis*-Linalool oxide (furanoid)	0.2	0.5
1080	1082	*p*-Mentha-2,4(8)-diene	---	0.1
1085	1086	Terpinolene	2.0	2.4
1086	1086	*trans*-Linalool oxide (furanoid)	0.3	0.6
1090	1091	*p*-Cymenene	tr	0.2
1099	1099	Linalool	0.6	1.1
1105	1104	Nonanal	---	0.1
1112	1113	*p*-Mentha-1,3,8-triene	---	0.1
1119	1119	*endo*-Fenchol	0.2	0.2
1124	1124	*cis-p*-Menth-2-en-1-ol	0.5	0.6
1127	1127	*allo*-Ocimene	0.1	0.1
1134	1135	2-Vinylanisole	---	0.1
1135	1136	Terpin-3-en-1-ol	0.1	0.1
1140	1140	*trans*-Pinocarveol	0.1	0.1
1142	1142	*trans-p*-Menth-2-en-1-ol	0.4	0.4
1146	1145	Camphor	tr	0.1
1154	1156	Camphene hydrate	0.2	0.1
1171	1170	Borneol	0.3	0.3
1178	1179	2-Isopropenyl-5-methyl-4-hexenal	---	0.1
1180	1180	Terpinen-4-ol	0.8	1.3
1186	1186	*p*-Cymen-8-ol	0.2	0.7
1194	1195	α-Terpineol	6.7	5.7
1196	1196	*cis*-Piperitol	0.1	0.2
1197	1197	Methyl chavicol (=Estragole)	0.2	0.3
1208	1208	*trans*-Piperitol	0.1	0.2
1250	1250	Chavicol	0.1	0.1
1253	1254	Piperitone	0.1	0.1
1256	1257	6-Undecanone	0.1	0.1
1277	1277	Phellandral	tr	0.1
1284	1285	Bornyl acetate	0.1	0.5
1290	1289	Thymol	tr	tr
1293	1293	2-Undecanone	0.1	0.2
1511	1512	γ-Cadinene	0.1	0.1
1517	1518	δ-Cadinene	0.2	0.2
1560	1562	(*E*)-Nerolidol	0.3	0.1
1561	1560	Dodecanoic acid	0.1	0.1
1575	1576	Spathulenol	0.1	0.2
1621	1582	Selin-6-en-4β-ol	0.2	0.1
1626	1628	1-*epi*-Cubenol	0.1	0.1
1642	1640	τ-Cadinol	0.4	0.3
1644	1644	τ-Muurolol	0.4	0.3
1647	1651	α-Muurolol (=δ-Cadinol)	0.1	0.1
1656	1655	α-Cadinol	0.8	0.7
1658	1658	Selin-11-en-4α-ol (=Kongol)	0.1	0.1
1766	1769	Benzyl benzoate	0.2	0.1
1870	1869	Benzyl salicylate	0.3	0.1
1962	1958	Palmitic acid	0.2	---
1996	1997	Isopimaradiene	---	0.2
2013	2007	18-*nor*-Abieta-8,11,13-triene	0.2	0.2
2041	2047	Thunbergol	0.4	0.1
2178	2180	Sandaracopimarinal	0.1	0.2
2237	2243	Isomiparinal	0.3	0.5
2245	2250	Palustral	0.3	0.1
2249	2253	Levopimarinal	0.5	0.2
2277	2274	Dehydroabietal	0.2	0.1
2322	2314	Abietal	0.2	0.1
2380	2372	Neoabietinal	0.1	tr
		Monoterpene hydrocarbons	71.8	73.3
		Oxygenated monoterpenoids	11.1	13.2
		Sesquiterpene hydrocarbons	0.3	0.2
		Oxygenated sesquiterpenoids	2.5	2.0
		Diterpenoids	2.4	1.6
		Benzenoid aromatics	0.8	0.7
		Others	10.8	8.3
		Total identified	99.7	99.2

RI_calc_ = Retention index values determined using the method of van den Dool and Kratz [26]. RI_db_ = Reference retention index values from the databases [27,28,29,30]. *P.c.l.* = *Pinus contorta* subsp. *latifolia*. tr = trace (<0.05%).

**Table 4:** Chemical composition (percent) of the leaf essential oils of *Pseudotsuga menziesii* var. *glauca* from southern Idaho.

RI_calc_	RI_db_	Compound	*P.m.g. *#1	*P.m.g. *#2	*P.m.g. *#3
883	884	Santene	1.0	1.3	1.7
916	918	Prenyl acetate	tr	tr	tr
921	923	Tricyclene	1.2	1.8	1.8
924	927	α-Thujene	tr	tr	0.1
933	933	α-Pinene	6.3	9.1	11.2
952	953	Camphene	15.0	15.2	19.5
973	972	Sabinene	0.5	0.2	0.5
980	978	β-Pinene	3.0	2.6	3.7
989	991	Myrcene	0.8	1.2	0.8
998	997	Ethyl hexanoate	---	---	tr
1006	1007	α-Phellandrene	0.1	0.1	0.1
1009	1009	δ-3-Carene	0.5	0.3	tr
1015	1015	1,4-Cineole	tr	tr	tr
1017	1018	α-Terpinene	0.2	0.1	0.1
1024	1025	*p*-Cymene	0.2	0.1	0.1
1030	1030	Limonene	3.9	5.4	4.0
1031	1031	β-Phellandrene	0.4	0.4	0.4
1033	1032	1,8-Cineole	tr	tr	tr
1035	1034	(*Z*)-β-Ocimene	0.1	tr	tr
1047	1046	(*E*)-β-Ocimene	5.4	2.3	0.7
1058	1058	γ-Terpinene	0.4	0.2	0.2
1087	1086	Terpinolene	1.5	1.1	1.0
1090	1090	Fenchone	tr	tr	tr
1091	1093	*p*-Cymenene	tr	tr	tr
1095	1094	Methyl benzoate	0.1	tr	---
1101	1101	Linalool	1.6	1.4	4.0
1108	1108	Maltol	0.1	---	---
1120	1120	*endo*-Fenchol	tr	0.1	tr
1124	1125	Methyl octanoate	---	tr	tr
1125	1124	*cis-p*-Menth-2-en-1-ol	0.1	0.1	tr
1127	1127	α-Campholenal	tr	tr	0.1
1141	1142	*trans-p*-Menth-2-en-1-ol	0.1	0.1	tr
1146	1145	Camphor	tr	0.1	0.1
1149	1149	*iso*-Pulegol	0.1	tr	tr
1152	1152	Citronellal	1.0	0.4	0.1
1156	1156	Camphene hydrate	0.8	2.0	0.7
1164	1165	*iso*-Borneol	tr	0.1	tr
1170	1170	Umbellulone	tr	tr	tr
1172	1173	Borneol	0.7	0.8	1.0
1179	1179	2-Isopropenyl-5-methyl-4-hexenal	tr	0.1	0.1
1181	1180	Terpinen-4-ol	1.4	0.7	0.8
1189	1189	*p*-Cymen-8-ol	tr	tr	tr
1192	1192	Methyl salicylate	0.8	tr	tr
1195	1195	α-Terpineol	0.8	1.0	0.9
1206	1206	Decanal	0.1	0.1	0.1
1209	1209	*trans*-Piperitol	tr	0.1	tr
1218	1219	*endo*-Fenchyl acetate	0.2	0.1	0.2
1230	1232	Citronellol	1.9	1.0	0.2
1231	1229	Thymyl methyl ether	---	---	tr
1238	1238	Neral	0.1	tr	---
1249	1248	Carvotanacetone	0.1	---	---
1254	1255	Geraniol	0.1	---	tr
1255	1254	Piperitone	1.4	4.1	3.2
1270	1268	Geranial	0.1	0.1	---
1287	1285	Bornyl acetate	40.2	41.1	38.7
1291	1287	Isobornyl acetate	0.3	0.1	0.2
1296	1296	*trans*-Pinocarvyl acetate	tr	tr	tr
1324	1326	Myrtenyl acetate	tr	tr	tr
1327	1327	4-Terpinenyl acetate	0.1	0.1	0.1
1335	1335	δ-Elemene	---	tr	tr
1350	1350	Citronellyl acetate	2.3	1.1	0.5
1352	1352	α-Longipinene	tr	---	0.1
1359	1361	Neryl acetate	tr	tr	tr
1376	1372	Longicyclene	---	---	tr
1377	1377	α-Copaene	---	---	tr
1380	1380	Geranyl acetate	2.7	0.5	0.2
1391	1390	trans-β-Elemene	0.1	0.1	0.1
1410	1411	Longifolene	0.1	0.2	0.3
1422	1424	(*E*)-β-Caryophyllene	tr	tr	tr
1434	1433	*trans*-α-Bergamotene	tr	0.1	tr
1456	1454	α-Humulene	tr	0.1	0.1
1462	1463	Tuberolactone	---	0.2	---
1472	1471	Massoia lactone	---	tr	---
1477	1478	γ-Muurolene	---	---	tr
1480	1482	α-Amorphene	tr	0.1	tr
1482	1483	Germacrene D	tr	tr	tr
1483	1482	γ-Himachalene	tr	0.1	tr
1490	1490	Prenyl benzoate	0.1	0.1	tr
1498	1497	α-Muurolene	---	---	tr
1505	1505	(*E*,*E*)-α-Farnesene	tr	---	tr
1518	1518	δ-Cadinene	---	tr	tr
1541	1541	(*E*)-α-Bisabolene	tr	0.1	0.1
1563	1564	(*E*)-Nerolidol	tr	tr	tr
1607	1601	Longiborneol (=Juniperol)	0.1	0.1	tr
1612	1613	Humulene epoxide II	tr	tr	tr
1630	1629	*iso*-Spathulenol	0.3	0.4	0.3
1653	1652	β-Himachalol	---	0.2	0.1
1655	1655	α-Cadinol	0.1	0.1	---
1773	1772	Benzyl benzoate	0.4	0.1	0.1
1874	1872	Benzyl salicylate	0.4	0.1	0.1
1930	1934	Cembrene	---	0.1	0.2
1998	1994	Manoyl oxide	---	tr	tr
2046	2038	Thunbergol	---	0.1	0.1
2059	2062	Manool	0.1	0.4	0.3
2150	2152	Abienol	---	0.1	0.1
		Monoterpene hydrocarbons	40.5	41.5	46.0
		Oxygenated monoterpenoids	56.0	55.0	51.1
		Sesquiterpene hydrocarbons	0.2	0.6	0.6
		Oxygenated sesquiterpenoids	0.5	0.7	0.4
		Diterpenoids	0.1	0.8	0.6
		Benzenoid aromatics	1.8	0.2	0.1
		Others	0.1	0.3	0.1
		Total identified	99.2	99.0	98.9

RI_calc_ = Retention index values determined using the method of van den Dool and Kratz [26]. RI_db_ = Reference retention index values from the databases [27,28,29,30]. *P.m.g.* = *Pseudotsuga menziesii* var. *glauca*. tr = trace (<0.05%).

**Table 5:** Chemical composition (percent) of the foliar essential oils of *Thuja plicata* from northern Idaho.

RI_calc_	RI_db_	Compound	*T.p. *#1	*T.p. *#2	*T.p. *#3	*T.p. *#4	*T.p. *#5
799	801	Hexanal	tr	tr	tr	tr	tr
844	842	Ethyl 2-methylbutyrate	0.2	0.1	0.2	0.3	0.2
847	846	(*Z*)-Salvene	tr	tr	tr	tr	tr
850	849	(2*E*)-Hexenal	0.1	0.1	0.1	0.1	0.1
851	853	(3*Z*)-Hexenol	0.1	tr	0.1	tr	tr
922	923	Tricyclene	tr	tr	tr	tr	tr
925	927	α-Thujene	0.4	0.1	0.3	0.2	0.2
933	933	α-Pinene	1.7	0.6	1.4	0.8	0.8
948	948	α-Fenchene	tr	tr	tr	tr	tr
950	950	Camphene	tr	tr	tr	tr	tr
972	972	Sabinene	3.0	1.7	2.4	2.2	1.4
978	978	β-Pinene	0.1	0.1	0.1	0.1	0.1
989	991	Myrcene	1.0	1.1	1.3	1.0	0.7
1016	1018	α-Terpinene	0.5	0.3	0.5	0.5	0.4
1023	1025	*p*-Cymene	0.6	0.4	0.5	0.4	0.6
1028	1030	Limonene	0.6	0.6	0.8	0.6	0.5
1030	1031	β-Phellandrene	tr	tr	tr	tr	tr
1034	1037	5-Methyl-(5*E*)-octen-2-one	0.1	0.1	0.1	0.1	0.2
1057	1058	γ-Terpinene	0.9	0.7	0.8	0.8	0.7
1070	1069	*cis*-Sabinene hydrate	0.4	0.3	0.3	0.3	0.3
1085	1086	Terpinolene	0.2	0.2	0.2	0.2	0.2
1094	1093	Ethyl sorbate	-	-	-	0.3	-
1099	1098	Perillene	tr	tr	tr	tr	tr
1105	1101	*trans*-Sabinene hydrate	0.3	-	-	-	-
1107	1105	α-Thujone	72.5	73.9	74.7	76.3	77.8
1119	1118	β-Thujone	7.4	8.2	6.1	6.6	5.2
1125	1124	*cis-p*-Menth-2-en-1-ol	0.2	0.2	0.2	0.2	0.1
1127	1126	α-Campholenal	tr	tr	tr	tr	tr
1143	1142	*trans-p*-Menth-2-en-1-ol	0.1	0.1	0.1	0.1	0.1
1146	1145	*trans*-Verbenol	0.1	0.1	0.1	0.1	0.1
1153	1153	*neo*-3-Thujanol	0.1	0.1	0.1	0.1	0.1
1158	1157	Sabina ketone	0.2	0.1	0.1	0.2	0.2
1176	1176	*trans*-Isopulegone	tr	0.1	tr	tr	0.1
1182	1180	Terpinen-4-ol	3.1	2.7	3.1	2.9	2.2
1188	1186	*p*-Cymen-8-ol	0.2	0.2	0.2	0.2	0.3
1195	1195	α-Terpineol	0.3	0.3	0.3	0.2	0.2
1198	1197	Methyl chavicol (=Estragole)	0.3	0.5	0.4	0.6	0.3
1202	1213	4-Hydroxy-α-thujone	0.6	0.4	0.4	0.5	1.0
1208	1208	Verbenone	0.1	0.1	0.1	0.1	0.2
1209	1209	*trans*-Piperitol	0.1	tr	tr	tr	tr
1219	1218	*trans*-Carveol	0.1	tr	tr	tr	0.1
1238	1238	Carvacryl methyl ether	tr	tr	0.1	tr	tr
1243	1242	Cuminal	tr	tr	tr	tr	0.1
1244	1246	Carvone	tr	tr	tr	0.1	0.1
1247	1250	Ethyl oct-(2*E*)-enoate	0.1	tr	tr	tr	tr
1249	1249	Carvotanacetone	0.1	0.1	0.1	0.1	0.1
1261	1260	*trans*-Sabinene hydrate acetate	0.1	0.1	0.1	0.1	0.1
1269	1259	Linalyl acetate	0.2	0.2	0.3	0.2	0.2
1288	1286	*trans*-Sabinyl acetate	0.1	0.1	0.1	0.1	0.1
1290	1293	3-Thujanyl acetate	0.2	0.1	0.2	0.1	0.1
1292	1290	Menthyl acetate	0.4	0.5	0.5	0.4	0.5
1299	1300	Carvacrol	0.1	0.1	0.1	0.1	0.1
1318	1322	Myrtenyl acetate	tr	0.1	0.1	0.1	0.1
1330	1327	*p*-Mentha-1,4-dien-7-ol	0.1	0.1	0.1	0.1	0.2
1337	1335	4-Terpinenyl acetate	0.1	0.1	0.1	0.1	0.1
1347	1346	α-Terpinyl acetate	0.3	0.3	0.3	0.2	0.2
1379	1378	Geranyl acetate	0.4	0.5	0.5	0.2	0.3
1401	1403	Methyl eugenol	tr	tr	tr	tr	tr
1447	1448	(*E*)-Cinnamyl acetate	tr	tr	tr	tr	tr
1582	1578	Furopelargone B	tr	-	0.1	-	-
1608	1607	β-Oplopenone	0.1	0.1	0.1	tr	0.1
1661	1659	α-Cadinol	tr	0.1	0.1	tr	tr
1740	1738	Oplopanone	0.1	0.1	0.1	0.1	0.1
1923	1926	Rimuene	0.2	0.4	0.1	0.2	0.3
1959	1962	Beyerene	0.6	0.7	0.6	0.3	0.4
2064	2058	Abietatriene	tr	0.1	tr	tr	tr
2174	^a^	15-Beyeren-19-ol methyl ether	tr	0.1	tr	tr	tr
2258	^b^	15-Beyeren-19-ol	0.2	0.7	0.1	0.2	0.3
2319	2315	*trans*-Totarol	tr	0.2	tr	tr	0.1
2336	^c^	15-Beyeren-19-ol acetate	1.0	1.3	1.1	1.1	1.6
		Monoterpene hydrocarbons	9.0	5.8	8.4	6.8	5.5
		Oxygenated monoterpenoids	87.7	89.0	88.1	89.6	90.2
		Sesquiterpene hydrocarbons	0.0	0.0	0.0	0.0	0.0
		Oxygenated sesquiterpenoids	0.2	0.3	0.3	0.1	0.2
		Diterpenoids	1.9	3.4	1.9	1.8	2.7
		Benzenoid aromatics	0.3	0.5	0.4	0.6	0.3
		Others	0.5	0.3	0.5	0.7	0.5
		Total identified	99.6	99.3	99.6	99.5	99.3

RI_calc_ = Retention index values determined using the method of van den Dool and Kratz [26]. RI_db_ = Reference retention index values from the databases [27,28,29,30]. *T.p.* = *Thuja plicata*. tr = trace (<0.05%). ^a^ The MS library match (NIST 20) is 91%, but a reference RI is not available. ^b^ The MS library match (NIST 20) is 87%, but a reference RI is not available. ^c^ The MS library match (NIST 20) is 92%, but a reference RI is not available.

**Table 6:** Enantiomeric distribution of chiral terpenoid components (percentage of each enantiomer) in gymnosperm essential oils from Idaho.

Compound	RT (min)	*A.l.l. *#1	*A.l.l. *#2	*P.e.e.*	*P.c.l. *#1	*P.c.l. *#2	*P.m.g. *#1	*P.m.g. *#2	*P.m.g. *#3	*T.p. *#1	*T.p. *#2	*T.p. *#3	*T.p. *#4	*T.p. *#5
(+)-α-Thujene	13.92	nd	nd	nd	nd	nd	nd	nd	nd	100.0	100.0	100.0	100.0	100.0
(−)-α-Thujene	13.99									0.0	0.0	0.0	0.0	0.0
(−)-α-Pinene	15.92	72.5	79.2	62.5	87.1	87.8	86.7	89.1	71.6	46.2	9.5	22.0	2.7	6.6
(+)-α-Pinene	16.40	27.5	20.8	37.5	12.9	12.2	13.3	10.9	28.4	53.8	90.5	78.0	97.3	93.4
(−)-Camphene	17.73	97.6	95.5	92.6	78.6	80.0	98.0	97.8	97.6	nd	nd	nd	nd	nd
(+)-Camphene	18.30	2.4	4.5	7.4	21.4	20.0	2.0	2.2	2.4					
(+)-Sabinene	19.74	nd	nd	nd	nd	nd	1.4	nd	3.2	100.0	100.0	100.0	100.0	100.0
(−)-Sabinene	20.60						98.6		96.8	0.0	0.0	0.0	0.0	0.0
(+)-β-Pinene	20.27	1.4	1.5	4.0	1.7	1.7	1.6	2.8	1.8	68.8	88.0	83.0	89.5	93.6
(−)-β-Pinene	20.62	98.6	98.5	96.0	98.3	98.4	98.4	97.2	98.2	31.2	12.0	17.0	10.5	6.4
(−)-α-Phellandrene	22.59	94.1	96.1	nd	nd	nd	nd	nd	nd	nd	nd	nd	nd	nd
(+)-α-Phellandrene	22.81	5.9	3.9											
(−)-Limonene	25.06	91.9	96.2	94.5	86.0	89.6	81.6	81.0	82.7	4.3	2.6	4.0	4.1	3.6
(+)-Limonene	25.99	8.1	3.8	5.5	14.0	10.4	18.4	19.0	17.3	95.7	97.4	96.0	95.9	96.4
(−)-β-Phellandrene	26.15	99.9	100.0	89.1	99.7	99.6	97.2	97.2	96.8	nd	nd	nd	nd	nd
(+)-β-Phellandrene	26.88	0.1	0.0	10.9	0.3	0.4	2.8	2.8	3.2					
(+)-*cis*-Sabinene hydrate	40.70	nd	nd	nd	nd	nd	nd	nd	nd	95.2	97.4	95.6	92.6	95.2
(−)-*cis*-Sabinene hydrate	41.25									4.8	2.6	4.4	7.4	4.8
(+)-α-Thujone	43.32	nd	nd	nd	nd	nd	nd	nd	nd	0.0	0.0	0.0	0.0	0.0
(−)-α-Thujone	44.88									100.0	100.0	100.0	100.0	100.0
(−)-Linalool	45.69	71.6	68.2	68.1	79.5	80.0	91.9	92.6	95.0	nd	nd	nd	nd	nd
(+)-Linalool	46.24	28.4	31.8	31.9	20.5	20.0	8.1	7.4	5.0					
(+)-β-Thujone	46.06	nd	nd	nd	nd	nd	nd	nd	nd	100.0	100.0	100.0	100.0	100.0
(−)-β-Thujone	---									0.0	0.0	0.0	0.0	0.0
(−)-Camphor	49.31	0.0	nd	98.0	nd	nd	nd	nd	nd	nd	nd	nd	nd	nd
(+)-Camphor	50.12	100.0		2.0										
(+)-Terpinen-4-ol	54.64	nd	30.6	44.2	44.0	43.5	32.1	36.0	35.0	74.2	72.5	73.4	73.7	73.9
(−)-Terpinen-4-ol	54.93		69.4	55.8	55.0	56.5	67.9	64.0	65.0	25.8	27.5	26.6	26.3	26.1
(−)-Borneol	58.59	100.0	100.0	100.0	nd	100.0	97.7	100.0	97.2	nd	nd	nd	nd	nd
(+)-Borneol	59.11	0.0	0.0	0.0		0.0	2.3	0.0	2.8					
(−)-Bornyl acetate	59.46	100.0	100.0	100.0	nd	100.0	100.0	100.0	100.0	nd	nd	nd	nd	nd
(+)-Bornyl acetate	---	0.0	0.0	0.0		0.0	0.0	0.0	0.0					
(−)-α-Terpineol	59.73	nd	nd	52.8	95.4	93.1	83.0	nd	82.8	29.7	36.1	30.5	29.1	32.4
(+)-α-Terpineol	60.58			47.2	4.6	6.9	17.0		17.2	70.3	63.9	69.5	70.9	67.6
(−)-Piperitone	62.74	79.0	82.3	nd	nd	nd	100.0	100.0	89.8	nd	nd	nd	nd	nd
(+)-Piperitone	63.22	21.0	17.7				0.0	0.0	10.2					
(+)-β-Bisabolene	75.34	16.2	40.7	nd	nd	nd	nd	nd	nd	nd	nd	nd	nd	nd
(−)-β-Bisabolene	75.51	83.8	59.3											
(−)-(*E*)-Nerolidol	83.40	79.3	nd	nd	nd	nd	nd	nd	nd	nd	nd	nd	nd	nd
(+)-(*E*)-Nerolidol	83.59	20.7												

RT = retention time, *A.l.l.* = *Abies lasiocarpa* var. *lasiocarpa*, *P.e.e.* = *Picea engelmannii* subsp. *engelmannii*, *P.c.l.* = *Pinus contorta* subsp. *latifolia*, *P.m.g.* = *Pseudotsuga menziesii* var. *glauca*, *T.p.* = *Thuja plicata*, nd = not detected.

**Table 7:** Collection and hydrodistillation details of *Abies lasiocarpa* var. *lasiocarpa* (*A.l.l.*) *Picea engelmannii* subsp. *engelmannii* (*P.e.e.*), *Pinus contorta* subsp. *latifolia* (*P.c.l.*), *Pseudotsuga menziesii* var. *glauca* (*P.m.g.*), and *Thuja plicata* (*T.p.*).

Tree Sample	Tree Characteristics	Coordinates, Elevation	Mass Foliage, g, Used for the Distillation	Essential Oil Yield, g, (% Yield)
*A.l.l.* #1	Mature, cone bearing	43°38′11″ N, 115°21′16″ W, 1699	124.94	2.013 (1.611%)
*A.l.l.* #2	Mature, cone bearing	43°39′26″ N, 115°24′28″ W, 2122 m	231.03	4.291 (1.857%)
*P.e.e.*	Mature, cone bearing	43°37′22″ N, 115°25′52″ W, 2372 m	200.64	1.830 (0.912%)
*P.c.l.* #1	Mature, cone bearing	43°37′56″ N, 115°19′30″ W, 1559 m	72.02	2.236 (3.105%)
*P.c.l.* #2	Mature, cone bearing	43°37′52″ N, 115°23′3″ W, 1999 m	88.74	1.510 (1.702%)
*P.m.g.* #1	Mature, cone bearing	43°36′40″ N, 115°17′2″ W, 1420 m	182.91	1.738 (0.950%)
*P.m.g.* #2	Mature, cone bearing	43°37′33″ N, 115°18′25″ W, 1492 m	208.22	3.045 (1.462%)
*P.m.g.* #3	Mature, cone bearing	43°37′46″ N, 115°22′0″ W, 1902 m	189.21	1.235 (0.653%)
*T.p.* #1	Mature, cone bearing	47°36′32″ N, 116°40′12″ W, 664 m	224.85	8.751 (3.892%)
*T.p.* #2	Sapling	47°36′32″ N, 116°40′12″ W, 664 m	62.32	0.618 (0.992%)
*T.p.* #3	Mature, cone bearing	47°36′29″ N, 116°40′10″ W, 662 m	263.26	6.973 (2.649%)
*T.p.* #4	Large tree, no apparent cones	47°36′1″ N, 116°39′30″ W, 722 m	99.10	4.695 (4.738%)
*T.p.* #5	Large tree, no apparent cones	47°35′52″ N, 116°39′26″ W, 720 m	89.47	3.960 (4.426%)

## Data Availability

All data are included in the article.

## Supplementary Materials

The following supporting information can be downloaded at: https://www.mdpi.com/article/10.3390/molecules28062477/s1, Table S1: Major components of *Pseudotsuga menziesii* from different geographical locations; Table S2: Comparison of the major components in the essential oils of *Abies lasiocarpa* var. *lasiocarpa* (*A.l.l.*), *Picea engelmanii* var. *engelmanii* (*P.e.e.*), *Pinus contorta* subsp. *latifolia* (*P.c.l.*), *Pseudotsuga menziesii* var. *glauca* (*P.m.g.*), and *Thuja plicata* (*T.p.*).

## Sample Availability

Samples of the essential oils are not available from the authors.
