# Supplementary material for: Gymnosperms of Idaho: Chemical Compositions and Enantiomeric Distributions of Essential Oils of Abies lasiocarpa, Picea engelmannii, Pinus contorta, Pseudotsuga menziesii, and Thuja plicata
Source: Molecules. 2023 Mar 8;28(6):2477. doi: 10.3390/molecules28062477 (PMC10057033; doi:10.3390/molecules28062477)
Supplement: Supplementary file 1 [file molecules-28-02477-s001.zip › molecules-2240090_Supplementary Table S1.pdf]

**Supplementary Table S1.** Major components of *Pseudotsuga menziesii* from different geographical locations.

| Compound | *P.m.g.* #1 (this work) | *P.m.g.* #2 (this work) | *P.m.g.* #3 (this work) | Washington #1 [34] | Washington #2 [16] | Yellowstone [16] | Washington #3 (APRC) | Argentina (APRC) | New Zealand #1 (APRC) |
|---|---|---|---|---|---|---|---|---|---|
| Santene | 1.0 | 1.3 | 1.7 | 0.0 | 0.0 | 1.8 | 0.0 | 0.1 | 0.0 |
| Tricyclene | 1.2 | 1.8 | 1.8 | 0.0 | 0.0 | 1.0 | 0.1 | 0.2 | 0.1 |
| α-Pinene | 6.3 | 9.1 | 11.2 | 6.2 | 6.4 | 7.0 | 12.8 | 9.8 | 13.7 |
| Camphene | 15.0 | 15.2 | 19.5 | 0.4 | 0.4 | 17.0 | 0.6 | 1.9 | 0.6 |
| Sabinene | 0.5 | 0.2 | 0.5 | 14.0 | 12.9 | 0.1 | 4.6 | 8.8 | 4.2 |
| β-Pinene | 3.0 | 2.6 | 3.7 | 25.8 | 25.5 | 2.5 | 37.4 | 29.5 | 32.7 |
| δ-3-Carene | 0.5 | 0.3 | 0.0 | 0.5 | 1.8 | 0.6 | 3.7 | 3.0 | 7.0 |
| Limonene | 3.9 | 5.4 | 4.0 | 0.8 | 1.6 | 6.6 | 1.8 | 2.2 | 2.2 |
| β-Phellandrene | 0.4 | 0.4 | 0.4 | 1.7 | 2.3 | 5.1 | 1.8 | 2.0 | 1.7 |
| (*Z*)-β-Ocimene | 0.1 | 0.0 | 0.0 | 0.0 | 0.0 | 0.0 | 0.6 | 0.4 | 0.2 |
| (*E*)-β-Ocimene | 5.4 | 2.3 | 0.7 | 0.0 | 0.1 | 0.0 | 0.4 | 0.3 | 0.4 |
| γ-Terpinene | 0.4 | 0.2 | 0.2 | 5.8 | 5.4 | 0.1 | 4.1 | 5.9 | 4.0 |
| Terpinolene | 1.5 | 1.1 | 1.0 | 16.8 | 14.6 | 1.2 | 9.1 | 12.6 | 9.4 |
| Camphene hydrate | 0.8 | 2.0 | 0.7 | 0.0 | 0.0 | 2.0 | 0.0 | 0.0 | 0.0 |
| Borneol | 0.7 | 0.8 | 1.0 | 0.0 | 0.0 | 1.3 | 0.1 | 0.0 | 0.0 |
| Terpinen-4-ol | 1.4 | 0.7 | 0.8 | 12.2 | 12.1 | 0.5 | 5.4 | 5.1 | 6.2 |
| α-Terpineol | 0.8 | 1.0 | 0.9 | 1.9 | 2.4 | 0.3 | 2.1 | 1.0 | 1.8 |
| Bornyl acetate | 40.2 | 41.1 | 38.7 | 0.1 | 0.2 | 44.7 | 0.2 | 1.1 | 0.2 |
| Citronellyl acetate | 2.3 | 1.1 | 0.5 | 1.9 | 1.2 | 0.1 | 3.7 | 3.1 | 3.8 |
| Geranyl acetate | 2.7 | 0.5 | 0.2 | 1.9 | 2.1 | 0.2 | 2.1 | 1.3 | 2.0 |

**Supplementary Table S1.** Continued.

| Compound | New Zealand #2 (APRC) | Arizona #1 [16] | New Mexico [16] | Austria (glauca) [35] | Bulgaria #1 [36] | Bulgaria #2 [37] | Arizona #2 [31] | Romania #1 [[38] | Romania #2 [38] | Serbia [39] |
|---|---|---|---|---|---|---|---|---|---|---|
| Santene | 0.0 | 2.1 | 1.7 | 0.0 | 0.0 | 0.0 | 0.0 | 0.0 | 0.0 | 0.0 |
| Tricyclene | 0.1 | 2.2 | 2.5 | 0.0 | 0.0 | 0.0 | 3.9 | 0.0 | 0.0 | 0.0 |
| α-Pinene | 13.6 | 9.7 | 4.5 | 6.2 | 6.5 | 0.0 | 15.8 | 3.9 | 9.2 | 4.9 |
| Camphene | 0.6 | 24.6 | 26.4 | 1.0 | 0.4 | 0.1 | 28.5 | 0.2 | 0.5 | 0.3 |
| Sabinene | 5.4 | 0.0 | 0.0 | 15.4 | 14.9 | 21.0 | 0.0 | 30.0 | 14.2 | 17.9 |
| β-Pinene | 27.4 | 8.1 | 3.2 | 13.4 | 20.8 | 39.5 | 14.5 | 7.2 | 30.3 | 15.2 |
| δ-3-Carene | 8.2 | 0.7 | 0.8 | 4.3 | 0.0 | 0.0 | 0.7 | 0.0 | 0.0 | 2.3 |
| Limonene | 2.6 | 5.0 | 6.0 | 3.2 | 1.1 | 0.4 | 12.2 | 1.7 | 2.5 | 2.8 |
| β-Phellandrene | 1.7 | 3.4 | 4.3 | 0.3 | 0.6 | 0.5 | 0.0 | 7.2 | 5.1 | 2.8 |
| (*Z*)-β-Ocimene | 0.2 | 0.0 | 0.0 | 0.1 | 0.3 | 7.1 | 1.4 | 0.1 | 0.1 | 0.0 |
| (*E*)-β-Ocimene | 0.3 | 0.0 | 0.5 | 0.3 | 0.4 | 0.3 | 0.0 | 0.4 | 0.2 | 0.0 |
| γ-Terpinene | 4.6 | 0.1 | 0.2 | 4.2 | 4.4 | 1.8 | 0.0 | 0.0 | 0.0 | 5.5 |
| Terpinolene | 11.2 | 1.0 | 1.2 | 7.3 | 11.8 | 20.2 | 1.7 | 23.2 | 12.7 | 22.7 |
| Camphene hydrate | 0.0 | 1.7 | 1.2 | 0.0 | 0.0 | 0.0 | 0.0 | 0.0 | 0.0 | 0.0 |
| Borneol | 0.0 | 2.2 | 1.5 | 0.3 | 0.0 | 0.1 | 0.5 | 0.1 | 0.2 | 0.0 |
| Terpinen-4-ol | 7.2 | 0.2 | 0.2 | 9.2 | 9.6 | 0.0 | 0.3 | 11.5 | 9.2 | 9.2 |
| α-Terpineol | 1.7 | 0.2 | 0.8 | 1.4 | 2.5 | 1.9 | 0.4 | 0.6 | 1.1 | 0.5 |
| Bornyl acetate | 0.3 | 32.4 | 28.9 | 3.1 | 0.0 | 0.3 | 0.0 | 0.0 | 0.0 | 0.0 |
| Citronellyl acetate | 3.3 | 0.1 | 0.4 | 5.1 | 1.5 | 0.5 | 0.0 | 0.1 | 2.1 | 5.5 |
| Geranyl acetate | 1.4 | 0.0 | 0.3 | 3.6 | 0.0 | 0.3 | 0.0 | 0.0 | 0.0 | 0.9 |
