# Supplementary material for: Gymnosperms of Idaho: Chemical Compositions and Enantiomeric Distributions of Essential Oils of Abies lasiocarpa, Picea engelmannii, Pinus contorta, Pseudotsuga menziesii, and Thuja plicata
Source: Molecules. 2023 Mar 8;28(6):2477. doi: 10.3390/molecules28062477 (PMC10057033; doi:10.3390/molecules28062477)
Supplement: Supplementary file 1 [file molecules-28-02477-s001.zip › molecules-2240090_Supplementary Table S2.pdf]

Supplementary Table S2. Comparison of the major components in the essential oils of *Abies lasiocarpa* var. *lasiocarpa* (*A.l.l.*), *Picea engelmanii* var. *engelmanii* (*P.e.e.*), *Pinus contorta* subsp. *latifolia* (*P.c.l.*), *Pseudotsuga menziesii* var. *glauca* (*P.m.g.*), and *Thuja plicata* (*T.p.*).

**Compound**	***A.l.l.*#1**	***A.l.l.*#2**	***P.m.g.*#1**	***P.m.g.*#2**	***P.m.g.*#3**	***P.e.e***	***P.c.l.*#1**	***P.c.l.*#2**	***T.p.*#1**	***T.p.*#2**	***T.p.*#3**	***T.p.*#4**	***T.p.*#5**
(2*E*)-Hexenal	0	0	0	0	0	0.4	7.1	5.3	0.1	0.1	0.1	0.1	0.1
α-Pinene	5.0	4.5	6.3	9.1	11.2	3.6	5.0	4.0	1.7	0.6	1.4	0.8	0.8
Camphene	**10.9**	**7.4**	**15.0**	**15.2**	**19.5**	6.0	0.4	0.4	tr	tr	tr	tr	tr
Sabinene	tr	0.1	0.5	0.2	0.5	0.3	0.3	0.4	3.0	1.7	2.4	2.2	1.4
β-Pinene	13.6	9.3	3.0	2.6	3.7	2.4	**27.0**	**20.3**	0.1	0.1	0.1	0.1	0.1
Myrcene	1.1	1.5	0.8	1.2	0.8	**11.7**	4.2	3.0	1.0	1.1	1.3	1.0	0.7
δ-3-Carene	tr	0.3	0.5	0.3	tr	3.7	3.6	11.0	-	-	-	-	-
Limonene	**20.3**	**34.6**	**3.9**	**5.4**	**4.0**	4.4	3.3	3.7	0.6	0.6	0.8	0.6	0.5
β-Phellandrene	6.7	7.1	0.4	0.4	0.4	4.3	**21.8**	**20.9**	tr	tr	tr	tr	tr
1,8-Cineole	-	-	tr	tr	tr	2.4	-	-	-	-	-	-	-
(*E*)-β-Ocimene	-	0.7	5.4	2.3	0.7	tr	0.1	0.6	-	-	-	-	-
Terpinolene	0.7	0.4	1.5	1.1	1.0	0.7	2.0	2.4	0.2	0.2	0.2	0.2	0.2
Linalool	0.5	0.4	1.6	1.4	4.0	1.2	0.6	1.1	-	-	-	-	-
α-Thujone	-	-	-	-	-	-	-	-	**72.5**	**73.9**	**74.7**	**76.3**	**77.8**
β-Thujone	-	-	-	-	-	-	-	-	**7.4**	**8.2**	**6.1**	**6.6**	**5.2**
Camphor	0.2	tr	tr	0.1	0.1	**22.8**	tr	0.1	-	-	-	-	-
Camphene hydrate	0.1	tr	0.8	2.0	0.7	6.0	0.2	0.1	-	-	-	-	-
Borneol	0.2	0.2	0.7	0.8	1.0	8.3	0.3	0.3	-	-	-	-	-
Terpinen-4-ol	0.2	0.2	1.4	0.7	0.8	0.6	0.8	1.3	3.1	2.7	3.1	2.9	2.2
α-Terpineol	0.4	0.3	0.8	1.0	0.9	2.8	6.7	5.7	0.3	0.3	0.3	0.2	0.2
Bornyl acetate	**24.7**	**18.5**	**40.2**	**41.1**	**38.7**	2.4	0.1	0.5	-	-	-	-	-
